# Assessment of Knowledge, Attitude, and Practice of Antibiotic Use among the Population of Boyolali, Indonesia: A Cross-Sectional Study

**DOI:** 10.3390/ijerph18168258

**Published:** 2021-08-04

**Authors:** Hidayah Karuniawati, Mohamed Azmi Ahmad Hassali, Sri Suryawati, Wan Ismahanisa Ismail, Taufik Taufik, Md. Sanower Hossain

**Affiliations:** 1 Discipline of Social and Administrative Pharmacy, School of Pharmaceutical Sciences, Universiti Sains Malaysia, Gelugor 11800, Pulau Pinang, Malaysia; azmihassali@usm.my; 2 Department of Pharmacology and Clinical Pharmacy, Faculty of Pharmacy, Universitas Muhammadiyah Surakarta, Surakarta 57102, Indonesia; 3 Faculty of Medicine, Public Health, and Nursing, Universitas Gadjah Mada, Yogyakarta 55281, Indonesia; suryawati@ugm.ac.id; 4 Faculty of Health Science, Universiti Teknology MARA, Cawangan Pulau Pinang Kampus Bertam, Kepala Batas 13200, Pulau Pinang, Malaysia; ismahanisa@uitm.edu.my; 5 Faculty of Psychology, Universitas Muhammadiyah Surakarta, Surakarta 57102, Indonesia; taufik@ums.ac.id; 6 Department of Biomedical Science, Kulliyyah of Allied Health Sciences, International Islamic University Malaysia, Kuantan 25200, Pahang, Malaysia; mshossainbge@gmail.com; 7 Faculty of Science, Sristy College of Tangail, Tangail 1900, Bangladesh

**Keywords:** antibiotics misuse, antibiotics resistance, knowledge, attitude, practice

## Abstract

Misuse and overuse of antibiotics are potential causes of the increasing prevalence of antibiotic resistance (ABR). Having information about the knowledge, attitude, and practices concerning antibiotics use by the public might help control ABR growth. Therefore, this cross-sectional study aimed to investigate the levels and associated factors of knowledge, attitude, and practice (KAP) of antibiotics use among the public. A questionnaire was designed and validated, which consisted of a total of 51 questions with four sections: demographics (6), knowledge (20), attitude (12), and practice (13) to measure KAP. Univariate analysis (using Mann–Whitney U and Kruskal–Wallis analysis) was applied to assess the differences in the mean scores of KAP. Linear regression analysis was performed to identify factors associated with KAP. Finally, using Spearman analysis we have examined the correlation between responses to the KAP. The sample size of this study was 575, with a 99.96% response rate. Regarding knowledge, 73.12% of respondents stated that antibiotics could be used to treat viral infections, and 63.35% of respondents answered that antibiotics could reduce fever. Concerning attitude, 50% of respondents had considered stopping taking antibiotics as soon as symptoms had disappeared. In analyzing practice, we found 40% of respondents obtained antibiotics from a pharmacy without a prescription from a physician, a nurse, or a midwife. Statistical analysis revealed that KAP about antibiotic use was significantly associated with gender, area of residence, level of education, and monthly income (*p* < 0.05). Our findings concluded that men, respondents with low income, those with low-level education, and those living in rural areas are more prone to excessive use of antibiotics without knowing the adverse effects of improper use and how it can contribute to high ABR. So it is urgently necessary to strengthen policies on antibiotics use, including drug provision, distribution, and sales. In addition, people with low KAP should be a priority consideration in education outreach initiatives.

## 1. Introduction

Antibiotic resistance (ABR) is a global threat that leads to treatment failure, increases in length of hospital stay, cost of care, morbidity, and mortality [1,2,3,4]. Additionally, ABR threatens human healthcare progress, agricultural production, and, ultimately, life expectancy. Among the causes of ABR, misuse and overuse of antibiotics directly affect ABR development [3,5,6], which occurs due to a lack of knowledge, careless attitudes, and incorrect beliefs of the public about antibiotics [6,7]. Patients’ belief in the remarkable effectiveness of antibiotics and their being universally efficacious against all ailments has resulted in overuse, which is one of the significant factors of the exponential growth of ABR [8,9,10]. For example, patients’ believed that antibiotics would help them in overcoming viral respiratory illnesses, such as the common cold [7,11,12,13]. Recently, the high prevalence of COVID-19 associated mucormycosis (CAM) has been witnessed by India, caused by resistant mucormycetes. Mucormycosis is a relatively rare type of fungal infection, but its prevalence has increased significantly due to the irrational use of broad-spectrum antibiotics in treating COVID-19 patients [14,15].

Respiratory and gastrointestinal diseases are common but are also commonly incorrectly managed by using antibiotics, and physicians needlessly write antibiotic prescriptions, increasing the chances of ABR prevalence [2,7,11,16]. A recent systematic review and meta-analysis has reported that antibiotics are used inappropriately by the general population, as seen in behaviours such as purchasing antibiotics from pharmacies without a prescription, demanding antibiotics from physicians, not following prescribed antibiotics, and using antibiotics as prophylaxis for non-indicated diseases [17]. An incomplete course of antibiotics—not completing the entire course of antibiotics prescribed by the physician—educates microbes to become resistant, probably leading to a higher rate of ABR development [18,19]. Another study conducted in the Philippines has reported that 78% of respondents (*N* = 218 with 69% having graduated high school) shared their antibiotics with others [20]. A dominant cause of the high prevalence of ABR is the availability of antibiotics over the counter, particularly in developing countries where the sale of antibiotics is not well documented and regulated. Therefore, the prevalence of self-medication with antibiotics is high for treating common symptoms, which do not require antibiotics. A review found that one third (33.7%) of the population practiced self-medication [21]. Barber et al. [10] reported that most patients do not necessarily require antibiotics for treatment. Patient pressure and the motivation of greater profit from selling more antibiotics further enhance the irrational use of antibiotics [22,23].

Several studies related to knowledge, attitude, and practice about antibiotics in the general population have been undertaken in a range of different countries [24,25,26,27,28,29]. These studies have shown that 57–79% of respondents said that antibiotics are good for treating infections caused by viruses [25,26,28,29], and 50% believed that antibiotics might shorten an upper respiratory tract infection [25]. Self-medication with antibiotics used as prophylaxis against infection was reported by more than 50% of respondents, and almost 42% of respondents discontinued antibiotics on the alleviation of symptoms [21,27,28].

Indonesia is overpopulated and is one of the top five most populous countries in the world and also experiences the misuse and overuse of antibiotics due to widespread inadequate knowledge and incorrect attitude [7,30,31,32]. Purchasing antibiotics without prescription and self-medication are highly prevalent in Indonesia. More than 50% of people have inaccurate knowledge about antibiotics, and believe that antibiotics cure viral infection and prevent the worsening of illness [31]. Self-medication as a tendency to try to relieve disease symptoms like those a patient has experienced already is high (48.5%). Respondents reported remembering which antibiotics had been suggested by their physician before and have purchased them again over the counter without getting a new prescription. Some respondents have reported that they have even followed relatives’ recommendations for prescriptions or their general medical advice (51.9%) to treat similar symptoms. They have justified this by saying that they can get rid of the disease and simultaneously save money. A few other Indonesian studies showed that respondents have usually self-medicated using the same antibiotics prescribed before for similar clinical symptoms to save time and money [32,33,34].

Antibiotic-resistant germs arise because of the irrational use of antibiotics that fails to meet the General Guidelines for the Use of Antibiotics. A study in Indonesia showed that 43% of *Escherichia coli* collected from community samples were resistant to the antibiotics ampicillin (34%), cotrimoxazole (29%), and chloramphenicol (25%) [35]. Therefore, the Indonesian government has implemented ABR control programs in 2015, such as Gerakan Masyarakat Cerdas Menggunakan Obat (GEMA CERMAT), to halt the misuse and overuse of antibiotics and improve the community’s rational use of antibiotics in hospitals and the community nationwide. One of the primary purposes of this program is to offer a series of activities to create antibiotics awareness, understanding, and good practice. As the government has implemented awareness programs nationwide, evaluating peoples’ current knowledge, attitude, and practice (KAP) towards antibiotics use is essential. Therefore, this study has assessed and evaluated the KAP of antibiotics use among the local community. The findings of this study might help the government to redesign policy and evaluate the implementation of ABR control programs.

## 2. Materials and Methods

### 2.1. Study Site

**Figure 1:**
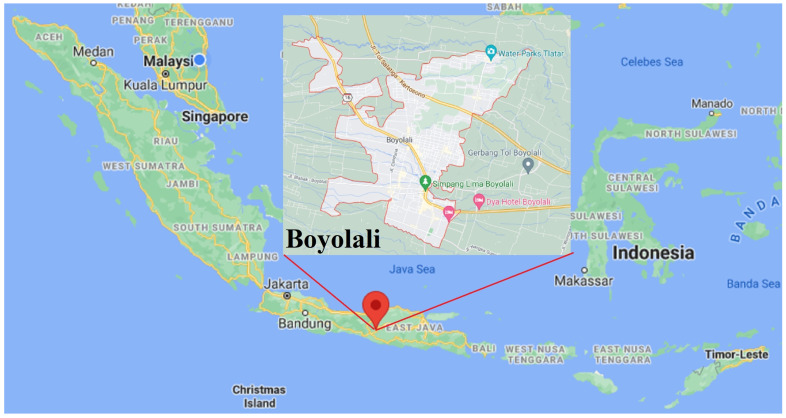
Data collection site of the cross-sectional study. Boyolali, Boyolali Regency, Central Java, Indonesia.

Indonesia is the largest and most populous country in Southeast Asia. It has 271.34 million people and 34 provinces with 416 regencies. The study area is located at Boyolali, Boyolali Regency, Central Java, Indonesia. It is in central Java, Indonesia, surrounded by the Grobogan and Semarang Regency to the north, Karanganyar, Sragen, and Sukoharjo Regency to the east, Klaten and Yogyakarta to the south, and Magelang and Semarang Regency to the west (Figure 1). Boyolali includes 19 subdistricts with 1.06 million populations. The average literacy of this region is 87.52% [36,37].

### 2.2. Study Design

The sample was chosen using the cluster sampling method following World Health Organization (WHO) guidelines [38,39]. Boyolali comprises 19 subdistricts, of which five subdistricts have been selected. One selection was made using judgmental sampling that comprised two subdistricts and another selection was made using random sampling that accounted for three subdistricts. A total of 20 clusters were identified from the selected subdistricts. The smallest administrative unit, the “village”, was considered a cluster. Judgmentally selected subdistricts cover the highest-income subdistrict (the capital city area), and the lowest-income subdistrict (rural areas) [38]. In each selected subdistrict, four villages were selected randomly, and 27–29 respondents in each village were involved. The first house visited in each cluster was randomly selected using existing lists of household names. After the first household visit, the second household was visited based on what was nearest to the first. The nearest household has been defined as the household which could be reached starting from the previously visited household in the shortest walking time. This cluster similarly included 29 respondents [39]. The same held for other clusters.

In this study, to select variables we used the theory of Health Belief Model (HBM). HBM suggests that a person’s health behavior is influenced by modifying factors (age, gender, ethnicity, level of education, socioeconomic status, and knowledge) and individual beliefs [40].

### 2.3. Sample Size

The sample size was determined using the Raosoft sample size calculator [41]. For a population of 1 million (Boyolali regency, Indonesia as per Department of Statistics, Indonesia) with a 95% confidence interval and 5% margin of error, the minimum number of estimated samples was 384. The sample size was increased by about 50% to minimize the weakness of cluster sampling and increase the representative sample [42]. The r^2^ was used to evaluate the effect size [43].

### 2.4. Data Instrument and Collection

A closed-ended cross-sectional quantitative survey was conducted in the Boyolali Regency, Indonesia, from January 2020 to March 2020. A validated self-administered questionnaire was used in this study [44]. The questionnaire was developed based on the literature reported [28,29,45,46,47], early individual interviews, and input from nine experts, including one clinical pharmacologist, two pharmacists, one community medicine specialist, two physicians, one clinical psychologist, one expert methodologist, and one expert epidemiologist. The validation phase consisted of face, content, and construct validity. Content validity ratio (CVR) and content validity index (CVI) were used to analyse content validity. Items which had CVR ≥ 0.78 and CVI > 80% were retained [48,49]. Construct validity was measured using exploratory factor analysis (EFA) and confirmatory factor analysis (CFA) [50,51,52]. Item analysis was employed for knowledge evaluation [48]. Reliability was evaluated with internal consistency reliability [53] and test–retest reliability in a two-week interval [54]. Validity and reliability were assessed using 407 respondents. Items’ difficulty and discrimination index in the knowledge section was found to be acceptable, with the Cronbach’s α and test–retest reliability being 0.827 and 0.713, respectively. Four factor-solutions emerged for the attitude and practice section with a cumulative contribution of 59.79% and 58.99%, respectively. The CFA result indicated acceptable fit indices for the proposed model (χ^2^/df ≤ 5, GFI > 0.90, RMSEA < 0.08); incremental fit (TLI > 0.90, CFI > 0.90); and parsimonious (PNFI 0.60–0.90) [55]. Every single factor in both the attitude and practice section had an acceptable range in internal consistency reliability, and test–retest reliability ranged from 0.717 to 0.898. Items that were found not to be valid and reliable were removed or revised [44]. The finalised questionnaire that had met the validity and reliability criteria was used to collect and measure data of KAP.

This questionnaire has included 20 questions relating to knowledge, 12 questions about attitudes, and 13 questions about practice. Based on the exploratory factor analysis (EFA), the knowledge section was divided into six domains, including “identification of antibiotics” (Q1–Q3), “role of antibiotics” (Q4–Q7), “antibiotics access” (Q8–Q11), “antibiotics misuse effect” (Q12–Q15), “side effect of antibiotics” (Q16–Q17), and “antibiotics use” (Q18–Q20). The attitude questions, based on the EFA result, were divided into four domains: “antibiotics resources” (Q1–Q5), “leftover” (Q6–Q8), “antibiotics use” (Q9–Q10), and “hopes about antibiotics” (Q11–Q12). The questions about practices were divided into four domains; “antibiotics resources” (Q1–Q7), “antibiotics recommendations” (Q8–Q9), “antibiotics use” (Q10–Q11), and “intention towards antibiotics use” (Q12–Q13).

Each correct response to a statement on knowledge of antibiotics was given a score of 1, whereas incorrect or “don’t know” responses were given a score of 0. The maximum possible score in the knowledge domain was 20. A five-point Likert scale ranging from 1 to 5 was used for scoring the attitude and practice questions. Score “1” was given for the least appropriate answer and “5” was given for the most appropriate response. Some of the questions were unfavorable questions, and the scores were inverted. The minimum and maximum possible scores for the attitude section were 12 and 60, respectively. The lowest possible score was 13, and the highest possible score was 65 for the practice section. The scores then were transformed to a scale ranging from 0 (worst possible score) to 100 (best possible score) with the formula (Equation (1)) [54,56]. The total score of <50%, 50-70%, and >70% were categorized as low, moderate, and high knowledge, attitude, and practice, respectively [28].

(1) $$\mathrm{Total}\;\mathrm{Score}\;(\%)=\frac{\mathrm{Obtained}\;\mathrm{score}-\mathrm{least}\;\mathrm{possible}\;\mathrm{score}}{\mathrm{Maximum}\;\mathrm{score}-\mathrm{least}\;\mathrm{possible}\;\mathrm{score}}\times 100$$

Data collection was carried out by a team of researchers supported by two assistants. Before starting the data collection, the people conducting the survey had received adequate briefing and training. They were informed that they were not allowed to be present while the respondents filled in the questionnaire. However, they have guided the respondents on how to fill in the questionnaire. The survey was conducted all day. The questionnaire was distributed and collected after it had been filled in, usually on the same day. The preferred respondents in this study were the heads of households. If the head of the household was absent at the time of the visit, the questionnaire was left at the home and then collected at the time agreed with the respondent or the oldest member of the household (above 17 years old), who filled in the questionnaire in the case of possible prolonged absence of the head of the household. If multiple families lived together with shared cooking and sleeping quarters, they were considered a single household and given one questionnaire [39]. The inclusion criteria were the general community having writing and communicating ability, having ever used antibiotics, and being willing to participate. Having health-related educational or occupational backgrounds was an exclusion criterion.

### 2.5. Ethical Issues

The study protocol received approval from the Medical and Health Research Ethics Committee of the Faculty of Medicine of Universitas Muhammadiyah Surakarta before the study was conducted (Reference No. 2063/B.1/KEPK-FKUMS/III/2019). The respondents were informed verbally about the nature of this study, and they were asked to sign an informed consent form as proof of participation in this study.

### 2.6. Data Analysis

Paper-based questionnaires were collected, and data were entered and analyzed using Statistical Package for the Social Sciences (SPSS) version 21 (International Business Machines Corporation, New York, United States). Descriptive statistics, univariate, and multivariate linear regression statistics have been used for data analysis.

Demographic characteristic variables and responses to the knowledge, attitude, and practice questions were analysed for descriptive statistics. Demographic data were reported as a percentage and mean ± SD (Standard Deviation). Responses from respondents in the form of “yes”, “no”, and “don’t know” on knowledge about antibiotics domain were assessed descriptively by calculating the percentage of each response. To simplify the description of attitudes towards antibiotics, the responses were combined into three categories, namely “disagree”, “doubtful”, and “agree”. Participants’ responses about the practice domain were assessed using the 5-Likert scale including “never”, “seldom”, “sometimes”, “often”, and “always”.

Because of non-normal distribution, the Mann–Whitney U test and Kruskal–Wallis test were run to assess differences in mean scores of KAP. Mann–Whitney U test was used for the independent variable with two groups (example: gender). Kruskal–Wallis was run for independent variables with more than two groups (example: level of education). Linear regression statistical analysis was used to determine the relationship between demographic characteristics of respondents and knowledge, attitude, and practice of antibiotics usage. Gender and area of residence were coded as a dichotomous indicator (‘female and living in an urban area’ were set as the reference); age was coded as a continuous indicator; marital status, educational level, and monthly income were coded as an interval scale. The Spearman correlation coefficient correlation test was administered to determine the correlation between knowledge and attitude, knowledge and practice, and attitude and practice. A statistically significant difference between groups was determined at the 95% confidence level (*p*-value < 0.05).

## 3. Results

A total of 575 questionnaires was distributed in the study area, of which 573 participants returned completed surveys. The recorded response rate was 99.6%. Respondents were male (50.6%), female (49.4%), those from a rural area (59.7%), married (77.7%), and ranged in age from 17 to 77 years (average: 37 ± 13). The highest percentage of respondents (42.4%) were senior high school graduates, and the lowest percentage of participants (1%) were post-graduates. A total of 77.3% of respondents had a monthly income below the regional minimum wage. So, most people belong to poor economic conditions. The demographic characteristics of respondents are described in Table 1.

### 3.1. Knowledge

Most respondents (46.9%) had moderate knowledge about antibiotics (Table 2). More than 50% of respondents answered correctly in three out of six domains: “identification of antibiotics” (Q1–Q3), “knowledge of antibiotics misuse effects” (Q12–Q15), and “knowledge about the side effects of antibiotics” (Q16–Q17). In the domain of “Knowledge of the role of antibiotics”, 73.12% of respondents answered that antibiotics could be used to treat infections due to viruses and 63.35% of respondents responded that antibiotics could reduce fever. In “antibiotics access”, more than 40% of respondents did not correctly answer the questions “antibiotics can be bought online” and “amoxicillin can be purchased at a pharmacy without a doctor’s prescription”. In the domain of knowledge of antibiotics use, more than 50% of respondents incorrectly answered the questions “antibiotics need to be stored in case of illness in the future” and “antibiotics should be stopped if the illness has improved” (Table 3).

The mean ± SD of knowledge was 52.98 ± 18.06% (Table 2). This score was significantly (*p* < 0.05) lower in men (51.47 ± 17.82%) than women (54.52 ± 18.21%) (Table 4). Respondents with no formal education–elementary school (49.86 ± 14.23%) and respondents with junior high school education (51.79 ± 15.97%) had lower knowledge scores than those with higher background education. In addition, the knowledge score was significantly (*p* < 0.001) lower among respondents who lived in rural (50.13 ± 14.54%) and respondents who had a monthly income <1,600,000 (51.37 ± 16.08%) (Table 4). Linear regression results showed that gender, age, area resident, educational level, and monthly income were significantly associated with antibiotics knowledge (Table 5).

### 3.2. Attitude

**Figure 2:**
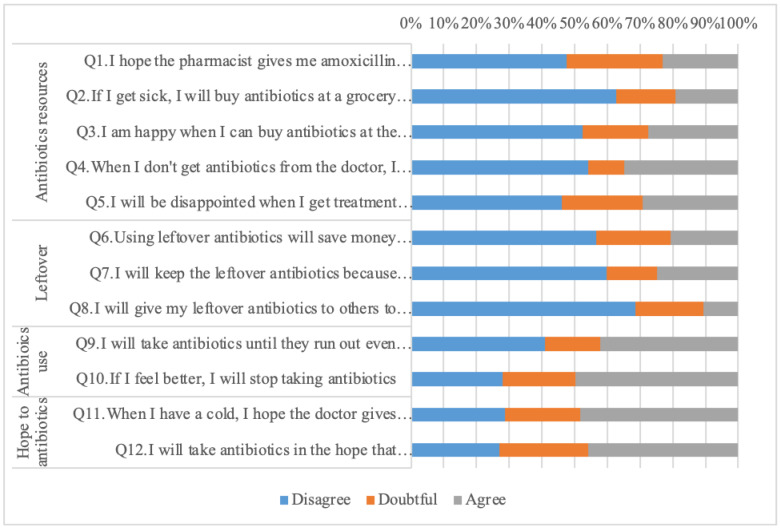
Responses patterns to questions about the attitude towards antibiotics. Data presented here is question-specific and not compared with any demographic characteristic.

Among the most important results on attitude (Figure 2), more than 45% of respondents considered taking antibiotics to speed up recovery from a cold, and respondents hoped the doctor would give them antibiotics. In a complementary way, 29% of respondents stated that they were disappointed if the doctor did not give them antibiotics, and 35% of respondents considered buying antibiotics at the pharmacy without a doctor’s prescription when they did not get antibiotics from the doctor. Additionally, 50% of respondents considered stopping taking antibiotics as soon as symptoms disappeared, and 25% of respondents stated they would keep leftover antibiotics for future treatment.

Scoring the data (0–100) showed that the average attitude score was 57.03 ± 15.43%, and 47.8% of respondents had a moderate attitude toward antibiotics (Table 2). The score was significantly (*p* < 0.05) lower in men (55.56 ± 14.96%) than in women (58.53 ± 15.77%). Respondents who lived in rural areas (54.67 ± 14.02%), respondents with no formal–elementary school education (52.19 ± 14.95%) and junior high school education (56.24 ± 13.40%), respondents with a monthly income <1,600,000 (51.37 ± 16.08%) also had significantly (*p* < 0.001) lower attitude score. Statistical analysis with linear regression revealed that antibiotics attitudes were significantly associated with gender, area of residence, level of education, and monthly income (Table 4 and Table 5).

### 3.3. Practice

**Figure 3:**
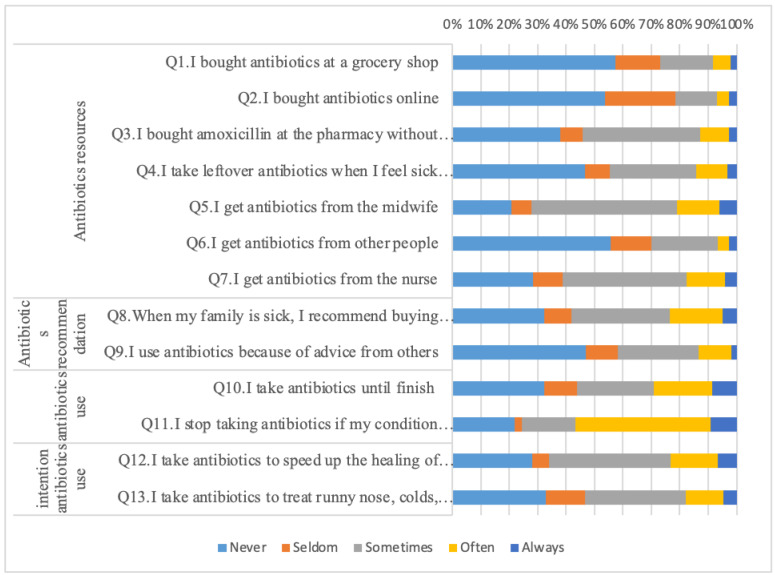
The response profile of antibiotics practices among the people. Data presented here is question-specific and not compared with any demographic characteristic.

Based on the questions about the practices of respondents in obtaining antibiotics, more than 50% of respondents never bought antibiotics from the grocery store or online and never obtained antibiotics from others. Nevertheless, 41% of respondents sometimes bought antibiotics from a pharmacy without a prescription, 43% of respondents obtained antibiotics from nurses, and 51% got antibiotics from midwives (Figure 3). Furthermore, as many as 31% of respondents occasionally took leftover antibiotics when experiencing sickness with the same symptoms. However, 35% of respondents sometimes recommended their family members should buy antibiotics when sick, 43% of respondents took antibiotics to speed up recovery from a cold, and 47% of respondents stopped taking antibiotics if their condition improved (Figure 3).

The data were transformed and put on a scale (0–100) to see the respondents’ general practices, and the average practice was 65.84 ± 17.23%. Practice scores were noted to be worse among men (64.19 ± 16.89%); people 50–60 years old (62.39 ± 18.62%); people ≥60 years old (59.15 ± 19.70%); rural people (63.01 ± 16.87%); widows/widowers (59.00 ± 20.18%); people with no formal education–elementary school (60.25 ± 16.36%); and people with a monthly income <1,600,000 (64.06 ± 16.63%). Respondent practices on antibiotics use were significantly associated with gender, area residence, level of education, and monthly income (*p* < 0.05). Females, urban people, people with higher levels of education, and people with higher monthly incomes had better practices in antibiotics use (Table 4 and 5).

### 3.4. Correlation between Knowledge, Attitude, and Practices of Respondents towards Antibiotics

Based on the results of the graphic scatterplot, the relationship between each variable of knowledge, attitude, and practices is linear so that the Spearman test could be continued. Furthermore, the results of correlation analysis using the Spearman correlation coefficient show a significantly (*p* = 0.001) positive correlation between each aspect of knowledge to attitude (0.488), attitude to practice (0.638), and knowledge to practices (0.442).

## 4. Discussion

This study has been intended to assess the KAP regarding antibiotics use among the general population of a regency in Indonesia using a validated questionnaire and following WHO guidelines. The findings have shown that each aspect of knowledge, attitude, and practice positively correlates. Our findings are consistent with previous studies [24,28]. It implies that the better one’s knowledge, the better the attitude, and the better the practice of antibiotics will be. Knowledge itself is not sufficient to change behavior but knowledge plays an important role in shaping beliefs and attitudes towards certain behaviors. There is always a correlation between knowledge, attitude, and practice concerning a particular subject. Inadequate knowledge about antibiotics also increases the overuse or misuse of antibiotics. We expected that the awareness program would have greater influence on antibiotic use among the public. However, this was not shown as happening, and our findings align with previous study results showing that respondents used antibiotics as self-medication to treat cold, fever, flu, cough, sore throat, and pain [7,28].

Although the average literacy rate in the study area is 87.52%, almost half (45.0–47.8%) of the respondents belong to the medium level of KAP about antibiotics. This is probably due to inadequate antibiotics information gained during schooling. For example, respondents had not learned in detail about the role of antibiotics, access to antibiotics, and how to use antibiotics rationally. Furthermore, the GEMA CERMAT method is carried out continuously but not on the same respondents because of the limited number of health workers (pharmacists). Even though a respondent might have received material about the prudent use of antibiotics, they might have forgotten this information due to dissemination not being carried out continuously.

A total of 46.9% of respondents to this study had a moderate level of antibiotics knowledge. Of the six domains, three domains regarding the respondents’ antibiotics knowledge showed respondents had retained information inaccurately. These areas of information cover the function of antibiotics, how to get antibiotics, and the correct use of antibiotics. These domains should be addressed as the focus of health education initiatives for the general community in the future. More than 63% of respondents assume that antibiotics can be used to treat virus infections and reduce fever. This is in line with the previous studies in China and the United States which revealed that 79% (675/854) of respondents in China and 57% (288/505) of respondents in the United States thought that antibiotics could cure viral infection [25,26]. In this study, respondents thought antibiotics could be bought online or purchased at a pharmacy without a doctor’s prescription. They also believed that antibiotics could be stopped if illness improved and stored for future use if needed. Another study also reported that more than 40% of respondents stopped antibiotics upon the alleviation of symptoms [27]. In line with a previous study [25], many respondents to our survey (45%) reported that they take antibiotics to speed up recovery from a cold and hope doctors will give them antibiotics. This belief indicates the respondents’ inadequate knowledge about the action of antibiotics and when antibiotics are prescribed by a physician. Unfortunately, sometimes physicians needlessly prescribed antibiotics [22]. Antimicrobial Stewardship Programs (ASPs) have been promoted and implemented by the Global Alliance for Infection in Surgery to minimize irrational antibiotic use and optimize antimicrobial usage by applying protocol pre-prophylaxis and recommending protocols for antimicrobial treatment of surgical infection. The interventions are both persuasive and obligatory, including audit and feedback, expert approval, compulsory order forms, educational materials, and outreach [57].

Insufficient knowledge of who is entitled to suggest antibiotics and where the antibiotics must be obtained (from a pharmacy with a doctor’s prescription) might promote self-medication. A previous study of the Indonesian population showed that most patients or consumers do not know that antibiotics should be obtained from the pharmacy with a prescription. Worryingly, more than 80% of community pharmacists sold antibiotics without a prescription [58], which is not expected as pharmacists know about antibiotics. In another study of the population of Saudi Arabia and Kuwait, 70% and 30% of respondents were taking self-prescribed antibiotics, respectively [27]. The same study also reported that more than 40% of respondents obtained antibiotics from the pharmacy without a doctor’s prescription. Surprisingly, more than 50% of respondents obtained antibiotics from midwives and nurses. According to Indonesian regulations, antibiotics can only be obtained in pharmacies with a doctor’s prescription [35].

Here, knowledge is not the central factor in the overuse of antibiotics; instead, attitude and practice are significant factors as pharmacists, nurses, and midwives are aware of antibiotics. Therefore, the government should implement strict regulations, and monitor self-medication, and control the sale of medicines not intended to be available over the counter. However, the reality is that our survey shows practices are not following the laws and regulations in Indonesia. Furthermore, as health workers, physicians, nurses, and midwives must provide education and health services within their competencies, further research is required regarding the factors that affect pharmacies’ administration of antibiotics without a doctor’s prescription, or a note from a nurse or midwife.

We have reported that people of high socioeconomic status, those with higher education levels, those living in urban areas, those with higher incomes, and those who are female are likely to have better knowledge, attitude, and practice of antibiotics use. A previous study has discovered that females, older respondents, those who were college-educated or had higher education, and those with a healthcare-related occupation or education were likely to have better knowledge, more appropriate attitudes, and better practice of antibiotics usage [24,59]. In contrast, inappropriate antibiotics were associated with males, younger respondents, and those who were married [25]. In addition, those who live in urban areas tended to be more health aware due to having better access to information and better exposure to community health-awareness programs [27].

The findings of this study would be helpful as the baseline for the future development of more effective public-education initiatives to improve knowledge, attitudes, and practices regarding antibiotic use among the general public. These findings could be an excellent platform for researchers to identify which areas need to be prioritized, create appropriate material for education, and choose the most suitable education methods so that the interventions given will be more focused, more on target, and more effective. The respondents’ knowledge, attitude, and practice show gaps regarding antibiotics role, antibiotics access, antibiotics use, and intention or reason for antibiotics use. Therefore, these areas should be made priority considerations in further educational programs. Males, those with low educational levels, living in rural areas, and having low income are more prone to overuse or misuse of antibiotics and should be the main target in subsequent educational programs.

Campaigns and education initiatives on the prudent use of antibiotics should always be carried out for the general population and health workers, including doctors, pharmacists, nurses, and midwives. There have been frequent seminars for pharmacists and doctors on the importance of the rational use of antibiotics. Meanwhile, there have not been many similar seminars for nurses and midwives, so it is necessary to conduct seminars on the prudent use of antibiotics for nurses and midwives. Moreover, controlling the distribution of antibiotics and applying drug regulation strictly for health workers requiring them to work within their competence and enforcing statutory regulations need to be more encouraged.

To the best of our knowledge, this is the first study in Indonesia that investigated the KAP towards antibiotics among the general population after the implementation of the national awareness program (GEMA CERMAT) intended for reducing ABR. This study has assessed and identified the levels and associated factors of KAP of antibiotics use among the public. The findings of this study might help the government to redesign policy and evaluate the implementation of Indonesian ABR control programs.

## 5. Strengths and Limitations of this Study

Respondents in this study included respondents who live in urban and rural areas with various ages, levels of income, and levels of education so that this study’s results could be seen as able to represent the whole population. Furthermore, the sampling of this study was conducted by visiting households one by one and talking face-to-face with the respondents. So, the response rate obtained was high (99.96%). Collecting face-to-face questionnaires allows researchers to double-check if the questionnaire has been completely filled in by respondents and to cross-check the questionnaire answers to minimize missing data. On the other hand, when filling out the questionnaire, some respondents were not taking antibiotics. So, they had to remember when they used antibiotics last, which may open a recall bias.

Furthermore, some respondents were not familiar with the word “antibiotics” but were more familiar with amoxicillin. Therefore, terminology might cause bias and make the score of the “identification of antibiotics” domain higher because when recruiting and identifying whether respondents met the inclusion criteria (ever used antibiotics), we changed the word “antibiotics” to “amoxicillin”. Hence, respondents answered the question quickly that “amoxicillin is antibiotics”.

Furthermore, because the self-administered questionnaire was conducted and the data were taken in the same household, there is a possibility that respondents who answered questions together caused each other to give similar responses. In addition, this study was only administered in one regency. These results may not be generalized to the whole Indonesian population because Indonesia consists of many islands/regencies with different ethnicities and cultures. Further study is suggested on this subject, looking at the different islands/regencies of Indonesia with the same/different demographic characteristics.

## 6. Conclusions

This survey has laid a foundation to better understand the knowledge, attitude, and practice of antibiotics after the implementation of the Gerakan Masyarakat Cerdas Menggunakan Obat (GEMA CERMAT) program by the Indonesian Government in 2015. To the best of our knowledge, this is the first survey conducted after an antibiotics’ awareness program was implemented nationwide. Still, we found that most respondents did not understand the correct function of antibiotics, antibiotics access, and the use of antibiotics. The findings of this study are essential as they provide valuable information to develop an intervention in public-health promotion to improve knowledge, attitudes, and practices towards antibiotics among the general public and will help policymakers tailor and design effective multifaceted interventions to improve prudent use of antibiotics in future.

Here, we suggest some interventions that could be immediately implemented nationwide to achieve the goal of sustainable healthcare and halt the spread of the ABR directly or indirectly. These are: (i) auditing the prescription of antibiotics; (ii) continuing public-education programs with the aim of not only increasing knowledge but also improving attitudes and practices in the use of antibiotics; (iii) targeting healthcare professionals (pharmacists, nurses, and midwives) in prohibiting the dispensation of antibiotics beyond allowed areas of authority; (iv) highlighting the role of healthcare professionals (pharmacists, nurses, and midwives) in health education and the promotion of appropriate antibiotics use by the public; and (v) controlling antibiotics distribution by applying strict antibiotics regulations.

## Figures and Tables

**Table 1:** Demographic characteristics of respondents.

Variables	Number (573)	Percentage (%)
Sex		
Male	290	50.6
Female	283	49.4
Age		
<20	39	6.8
20–39	286	49.9
40–59	208	36.3
≥60	40	7.0
Marital status		
Married	445	77.7
Single	105	18.3
Widow/widower/divorced	23	4
Level of education		
No formal education to elementary school	148	25.8
Junior high school	126	22.0
Senior high school	243	42.4
Diploma-graduate	50	8.8
Postgraduate	6	1.0
Area residence		
Urban	231	40.3
Rural	342	59.7
Monthly income (IDR)		
<1,600,000	443	77.3
1,600,000–3,000,000	90	15.7
>3,000,000	40	7.0

IDR = Indonesian Rupiah.

**Table 2:** The number of questions, range, score, and level of knowledge, attitude, and practice.

Variables	Number of Questions	Range of Score	Total Score (%) (Mean ± SD)	Level (%), *N* = 573		
				Low (<50%)	Moderate (50–70%)	High (>70%)
Knowledge	20	0–100	52.98 ± 18.06	38.9	46.9	14.2
Attitude	12	0–100	57.03 ± 15.43	31.6	47.8	20.6
Practice	13	0–100	65.84 ± 17.23	16.6	45.0	38.4

**Table 3:** Responses to the questionnaire on antibiotic knowledge (*N* = 573).

Domain	Statements		Respondents’ Answer *N* (%)		
		Expected Ideal Response	Correct	Incorrect	Don’t know
Identification of antibiotics	Q1. Amoxicillin is antibiotic	Yes	404 (70.51)	93 (16.23)	76 (13.26)
	Q2. Supertetra is antibiotic	Yes	341 (59.51)	145 (25.31)	87 (15.18)
	Q3. Paracetamol is antibiotic	No	236 (41.19)	246 (42.93)	91 (15.88)
Knowledge on the role of antibiotics	Q4. Antibiotics can kill bacteria	Yes	523 (91.27)	15 (2.62)	35 (6.11)
	Q5. Antibiotics can be used to treat infections due to viruses	No	74 (12.91)	419 (73.12)	80 (13.96)
	Q6. Colds and flu can be cured without antibiotics	Yes	413 (72.08)	113 (19.72)	47 (8.20)
	Q7. Antibiotics can reduce fever	No	136 (23.73)	363 (63.35)	74 (12.91)
Knowledge on antibiotics access	Q8. Antibiotics can be bought online	No	199 (34.73)	248 (43.28)	126 (21.99)
	Q9. Antibiotics from other people may be taken	No	379 (66.14)	110 (19.20)	84 (14.66)
	Q10. Amoxicillin can be purchased at a pharmacy without a doctor’s prescription	No	194 (33.86)	305 (53.23)	74 (12.91)
	Q11. Antibiotics can be purchased at the grocery shop	No	342 (59.69)	162 (28.27)	69 (12.04)
Knowledge on antibiotics misuse effect	Q12. Inappropriate use of antibiotics will cause antibiotic resistance	Yes	388 (67.71)	89 (15.53)	96 (16.75)
	Q13. Inappropriate use of antibiotics will cause these antibiotics not to be usable later	Yes	314 (54.80)	115 (20.07)	144 (25.13)
	Q14. Inappropriate use of antibiotics can cause more severe illness	Yes	370 (64.57)	103 (17.98)	100 (17.45)
	Q15. Inappropriate use of antibiotics increases costs	Yes	295 (51.48)	175 (30.54)	103 (17.98)
Knowledge on side effect of antibiotics	Q16. Antibiotics can cause allergic reactions, such as redness of the skin	Yes	391 (68.24)	86 (15.01)	96 (16.75)
	Q17. Antibiotics can kill good bacteria in the intestines	Yes	294 (51.31)	119 (20.77)	160 (27.92)
Knowledge on antibiotics use	Q18.Antibiotics need to be stored in case of illness in the future	No	208 (36.30)	309 (53.93)	56 (9.77)
	Q19. Leftover Antibiotics can be used again if sick	No	353 (61.61)	163 (28.45)	57 (9.95)
	Q20. Antibiotics can be stopped if the illness has improved	No	212 (37.00)	306 (53.40)	55 (9.60)

**Table 4:** Association of demographic characteristics and knowledge, attitude, and practice based on univariate analysis.

Variables	*N*	Knowledge (%)		Attitude (%)		Practice (%)	
		Mean ± SD	*p*-Value	Mean ± SD	*p*-Value	Mean ± SD	*p*-Value
Gender *							
Men	290	51.47 ± 17.82	0.040	55.56 ± 14.96	0.009	64.19 ± 16.89	0.012
Women	283	54.52 ± 18.21		58.53 ± 15.77		67.53 ± 17.43	
Age (year) **							
<20	39	53.59 ± 18.85	0.067	59.26 ± 14.18	0.133	67.56 ± 16.92	0.016
20–29	132	50.19 ± 17.74		58.12 ± 14.88		67.33 ± 17.94	
30–39	154	53.25 ± 18.50		56.89 ± 13.95		65.58 ± 15.28	
40–49	126	55.32 ± 19.27		58.66 ± 16.03		68.44 ± 16.42	
50–60	82	55.55 ± 17.21		53.82 ± 17.18		62.39 ±18.62	
≥60	40	47.88 ± 12.19		53.20 ± 17.17		59.15 ± 19.70	
Area Residence *							
Rural	342	50.13 ± 14.54	<0.001	54.67 ± 14.02	<0.001	63.01 ± 16.87	<0.001
Urban	231	57.19 ± 21.64		60.52 ± 16.72		70.04 ± 16.93	
Marital status **							
Married	437	53.00 ± 17.89	0.910	56.72 ± 15.65	0.211	65.32 ± 17.01	0.019
Single	113	53.32 ± 19.20		58.99 ± 13.63		69.26 ± 16.93	
Widow/Widower	23	50.87 ± 16.21		53.22 ± 18.69		59.00 ± 20.18	
Educational Level **							
No formal–elementary school	148	49.86 ± 14.23	0.003	52.19 ± 14.95	<0.001	60.25 ± 16.36	<0.001
Junior	126	51.79 ± 15.97		56.24 ± 13.40		66.64 ± 14.97	
Senior	243	53.68 ± 19.38		58.37 ± 15.48		66.73 ± 17.98	
Diploma-graduate	50	59.20 ± 23.35		64.78 ± 16.08		73.92 ± 16.18	
Post-graduate	6	74.17 ± 14.30		74.00 ±17.14		83.67 ± 16.06	
Monthly income **							
<1,600,000	443	51.37 ± 16.08	<0.001	55.02 ± 14.19	<0.001	64.06 ± 16.63	<0.001
1,600,000–3,000,000	90	57.44 ± 22.50		62.47 ± 17.59		70.61 ± 18.10	
>3,000,000	40	60.75 ± 23.74		66.95 ± 16.96		74.88 ± 17.23	

* Mann–Whitney; ** Kruskal–Wallis test.

**Table 5:** Linear regression models for knowledge, attitude, and practice domain (*N* = 573).

Variables	Knowledge			Attitude			Practices		
	Regression Coefficient	Standard Error	*p*-Value	Regression Coefficient	Standard Error	*p*-Value	Regression Coefficient	Standard Error	*p*-Value
Intercept	39.163	3.338	<0.001	49.661	2.793	<0.001	59.430	3.147	<0.001
Gender	4.272	1.492	0.004	3.594	1.249	0.004	3.799	1.407	0.007
Age	0.137	0.063	0.030	−0.015	0.053	0.781	−0.049	0.060	0.409
Area residence	5.803	1.537	<0.001	4.006	1.286	0.002	5.471	1.449	<0.001
Marital status	−0.241	1.437	0.867	−0.631	1.202	0.600	−0.758	1.355	0.576
Education level	2.392	0.836	0.004	2.423	0.699	0.001	2.413	0.788	0.002
Monthly income	3.199	1.341	0.017	4.668	1.122	<0.001	3.709	1.264	0.003

## Data Availability

Data is contained within the article or Supplementary Material.

## Supplementary Materials

The following materials are available online at https://www.mdpi.com/article/10.3390/ijerph18168258/s1, Table S1. Knowledge, attitude, and practices towards antibiotics questionnaire (KAPAQ).
