# Supplementary material for: Assessment of Knowledge, Attitude, and Practice of Antibiotic Use among the Population of Boyolali, Indonesia: A Cross-Sectional Study
Source: Int J Environ Res Public Health. 2021 Aug 4;18(16):8258. doi: 10.3390/ijerph18168258 (PMC8394957; doi:10.3390/ijerph18168258)
Supplement: Supplementary file 1 [file ijerph-18-08258-s001.zip › ijerph-1291016-supplementary.pdf]

International Journal of
Environmental Research and Public Health

MDPI

Supplementary Table S1

KNOWLEDGE, ATTITUDE, AND PRACTICES TOWARDS ANTIBIOTICS QUESTIONNAIRE (KAPAQ)

Part I. Knowledge of antibiotics

Answer the following statement by marking (√) with the answer that you think is correct.

No	Statement	Yes	No	Do not know
1	Amoxicillin is an antibiotic.			
2	Supertetra® is antibiotic.			
3	Paracetamol is antibiotic.			
4	Antibiotics are used to kill bacteria.			
5	Antibiotics can be used to treat infections due to viruses.			
6	Colds and flu can be cured without antibiotics.			
7	Antibiotics can reduce fever.			
8	Antibiotics can be bought online.			
9	Antibiotics from other people may be taken.			
10	Amoxicillin can be purchased at a pharmacy without a doctor's prescription.			
11	Antibiotics can be purchased at the grocery shop.			
12	Inappropriate use of antibiotics will cause antibiotic resistance.			
13	inappropriate use of antibiotics will cause these antibiotics cannot be used later.			
14	Inappropriate use of antibiotics can cause more severe illness.			
15	Inappropriate use of antibiotics increases costs.			
16	Antibiotics can cause allergic reactions such as redness of the skin.			
17	Antibiotics can kill good bacteria in the intestines.			
18	Antibiotics need to be stored in case of illness in the future.			
19	Antibiotics leftover can be used again if sick.			
20	Antibiotics can be stopped if the ill has improved.			

Part II Attitude towards antibiotics

Please mark (√) in the box that best suits you with SD choice: strongly disagree, D: disagree, N: doubtful, A: agree, SA: strongly agree

No	Statement	SD	D	N	A	SA
1	I hope the pharmacist gives me amoxicillin when I buy it at the pharmacy without a doctor's prescription					
2	If I get sick, I will buy antibiotics at a grocery shop					
3	I am happy when I can buy antibiotics at the pharmacy without a physician's prescription					
4	When I do not get antibiotics from the doctor, I will buy antibiotics at the pharmacy					
5	I will be disappointed when I get treatment but I do not get antibiotics					

No	Statement	SD	D	N	A	SA
6	Using leftover antibiotics will save money because I do not need to see a doctor					
7	I will keep the leftover antibiotics because they are useful in the future					
8	I will give my leftover antibiotics to others to help cure him					
9	I will take antibiotics until they run out even though my sick has improved					
10	If I feel better, I will stop taking antibiotics					
11	When I have a cold, I hope the doctor gives me antibiotics					
12	I will take antibiotics in the hope that antibiotics can speed up the healing of my cold					

Part III Practice using antibiotics

For each statement, please mark (√) that suits you (N: never, SD: seldom, S: sometimes, O: often, A: always).

No	Statement	N	SD	S	O	A
1	I bought antibiotics at a grocery shop					
2	I bought antibiotics online					
3	I bought amoxicillin at the pharmacy without a doctor's prescription					
4	I take leftover antibiotics when I feel sick with the same symptoms					
5	I get antibiotics from the midwife					
6	I get antibiotics from other people					
7	I get antibiotics from the nurse					
8	When my family is sick, I recommend buying antibiotics					
9	I use antibiotics because of advice from others					
10	I take antibiotics until finish					
11	I stop taking antibiotics if my condition improves					
12	I take antibiotics to speed up the healing of my cold					
13	I take antibiotics to treat runny nose, colds, tired aches, rheumatic pain, and flu					

Part IV: Demographic information

Answer the following questions by filling in the blanks (no. 1 to 3) and marking (√) in the box provided following your conditions.

1	Name	
2	Age	
3	address/mobile number	
4	Gender	☐ Male ☐ Female
5	Level education	☐ No formal education<br>☐ Elementary school<br>☐ Junior high school<br>☐ Senior high school<br>☐ Diploma<br>☐ Undergraduate<br>☐ Postgraduate
6	Monthly income	☐ <IDR.1.600.000

		☐ IDR.1.600.000-Rp.3.000.000<br>☐ >IDR.3.000.000
7	Marital Status	☐ Married<br>☐ Single<br>☐ Widow/widower

IDR: Indonesian rupiah
